# Pyruvate Kinase M2 Knockdown Suppresses Migration, Invasion, and Epithelial-Mesenchymal Transition of Gastric Carcinoma via Hypoxia-Inducible Factor Alpha/B-Cell Lymphoma 6 Pathway

**DOI:** 10.1155/2020/7467104

**Published:** 2020-12-09

**Authors:** Ning Li, Dandan Meng, Yue Xu, Ling Gao, Fengqian Shen, Xiaojing Tie, Yan Zhang, Zhenying Yi, Wenjie Shen, Zonglan Liu, Zhiqiao Xu

**Affiliations:** ^1^Tumor Diagnosis and Treatment Center of Kaifeng Central Hospital, Kaifeng, 475001 Henan, China; ^2^Analysis Department, Central Hospital of Kaifeng, Kaifeng, 475001 Henan, China

## Abstract

Gastric carcinoma is a common malignant cancer. Pyruvate kinase M2 (PKM2) is highly expressed in cancers, including gastric carcinoma. However, its function and molecular mechanism in gastric carcinoma remains unclear. Here, we aimed to explore the function and the underlying mechanism of PKM2 on malignant phenotypes in gastric carcinoma. In this study, the mRNA levels and protein levels of PKM2 in gastric carcinoma cell lines and normal gastric mucosa epithelial cell lines were detected using quantitative real-time PCR and western blot, respectively. PKM2 was downregulated by siRNA transfection. HIF-1*α* or BCL-6 was upregulated by corresponding overexpression plasmid. Cell viability was detected using CCK-8 assay. Cell invasion and migration were determined using transwell assay. Higher expression of PKM2 was observed in human gastric carcinoma cell lines MKN-45 and SGC-7901 than in the normal gastric mucosa epithelial cell line GES-1. PKM2 knockdown suppressed cancer cell invasion and migration and inhibited the epithelial-mesenchymal transition (EMT) phenotype by inhibiting E-cadherin and promoting vimentin and N-cadherin expression. Also, we observed that PKM2 knockdown suppressed the hypoxia-inducible factor alpha (HIF-1*α*) and B-cell lymphoma 6 (BCL-6) signaling pathway. HIF-1*α* overexpression reversed the function of PKM2 silencing on cell invasion, migration, EMT, and BCL-6 expression. BCL-6 overexpression also reversed the function of PKM2 silencing on cell invasion, migration, and EMT but did not affect HIF-1*α* expression. Taken together, data from our study suggest that PKM2 knockdown impeded cell migration, invasion, and EMT of gastric carcinoma cells via the HIF-1*α*/BCL-6 pathway.

## 1. Introduction

Gastric carcinoma is a gastrointestinal cancer and the third leading cause of cancer deaths worldwide [1, 2]. Despite a significant worldwide decline, gastric cancer remains the 5th most commonly diagnosed cancer in the world, and the incidence remains high in East Asia [3]. In 2018, there were about one million new diagnoses of gastric carcinoma and 783,000 gastric carcinoma-related deaths worldwide [4]. Gastric cancer accounts for 8.3% of all cancer deaths. Surgical treatment is the most effective therapy, curing 90% of patients with gastric cancer. However, most patients with gastric cancer are diagnosed in its advanced stage, leading to relatively low therapeutic efficacy [5]. The average 5-year survival rate for gastric cancer is 31%, but highly variable based on stage during surgical intervention [4]. The 5-year survival rate can be 94% in early stage treated with surgery, and 18% in the late stage treated with surgery [4].

Epithelial-mesenchymal transition (EMT) is the process of acquisition of the mesenchymal properties by epithelial, which commonly occurred in gastric cancer [6]. During EMT, the adhesion protein was decreased, and the intercellular contacts are disrupted. Cell motility was thus enhanced, leading to the release of cells from the parent epithelial tissue [6]. This resulted in tumor cell invasion and migration, allowing metastatic progression to proceed [6]. In the process of EMT, the expression of epithelial biomarkers (such as E-cadherin) was declined, and the mesenchymal biomarkers (such as N-cadherin, fibronectin, and vimentin) were elevated. It was reported that the EMT process is essential for tumor cell invasion and migration and thus accounts for the malignancy of gastric cancer [7]. Thus, it is urgent to attenuate the progression of gastric cancer by suppressing the invasion, migration, and EMT.

Metabolic reprogramming is common in gastric cancer, and the abnormal glycolysis in gastric cancer confers the invasion, metastasis, and drug resistance [8, 9]. Pyruvate kinase, a rate-limiting enzyme, is an important regulator of glycolysis [10]. Pyruvate kinase has four isoforms: pyruvate kinase M1 (PKM1) is expressed in the muscle, brain, and heart; PKM2 is upregulated in tumor cells and embryos; PKR is found in red blood cells; and PKL is the liver isoform [11]. PKM2 also is expressed in normal tissues of the colon, lung, kidney, thyroid, bladder, and liver [12, 13]. As a glycolytic enzyme, PKM2 is closely associated with tumor cell biology, including cell growth, migration, invasion, and metabolism [14–16]. Almost all tumor cells express PKM2, which promotes cancer cell growth and proliferation by increasing glycolysis and anabolic metabolism [12, 15, 17, 18].

Hypoxia is a prominent feature of tumor microenvironment. Hypoxia-inducible factor 1 (HIF-1) is a critical transcriptional factor of the mammalian response to hypoxia. It is composed of HIF-1*α* and HIF-1*β* subunits. Activation HIF-1*α* is involved in metabolic reprogramming, motility, metastasis, stem cell maintenance, angiogenesis, invasion, extracellular matrix remodeling, immune evasion, and EMT [19]. HIF-1*α* is overexpressed in hypoxic tumor tissues, including gastric cancer, and activates the transcription of many oncogenes, leading to the promotion of tumor growth [20, 21]. It was reported that PKM2 was a target gene of HIF-1*α*. Its transcription can be induced in response to hypoxia and regulated by HIF-1*α* [22]. Intriguingly, PKM2 interacts with HIF-1*α* physically and functionally to stimulate the binding of HIF-1*α* at target genes and promotes HIF-1*α* transcriptional activity in human cancer cells [22]. This evidence suggested a positive feedback loop between HIF-1*α* and PKM2.

B-cell lymphoma gene 6 (BCL-6) belongs to the BTB-POZ family and is necessary for the development of a number of immune cells, including germinal center B cells and CD4^+^ T follicular helper cells. Many genes that involved in cell cycle, cell death, and cell differentiation were regulated by BCL-6 [23]. In T helper cells exposed to interleukin 2, numerous genes in the glycolysis pathway were repressed by Bcl-6, including Slc2a1, Slc2a3, as well as the rate-limiting enzymes HK2 and PKM2 [23]. BCL-6 also serves as an oncogene in cancers. BCL-6 was overexpressed in breast cancer tissues and was positively associated with the expression of p53 and HIF-1*α* [24]. In gastric carcinoma, BCL-6 was overexpressed in human gastric carcinoma tissues compared with adjacent nontumor tissues [25]. The overexpressed BCL-6 in gastric carcinoma tissues was positively correlated with a malignant phenotype in patients with gastric carcinoma [25]. In gastric carcinoma cell lines, BCL-6 was also upregulated. BCL-6 inhibition by miR-519d-3p suppressed cell proliferation, invasion, cell cycle, and EMT in gastric carcinoma cells [25].

Collectively, we hypothesize that PKM2 expression was upregulated in human gastric carcinoma. The upregulated expression of PKM2 might promote the malignant phenotypes of gastric carcinoma. HIF-1*α* and BCL-6 might participate in the function of PKM2. In this study, we found that PKM2 knockdown suppressed invasion, migration, and EMT in human gastric carcinoma cells. Moreover, we confirmed that the HIF-1*α*/BCL-6 signal pathway was involved in the function of PKM2. This study provided a potential molecular target for the gastric carcinoma therapy based on PKM2.

## 2. Materials and Methods

### 2.1. Cell Culture and Transfection

The human gastric carcinoma cell lines MKN-45 and SGC-7901, as well as human normal gastric mucosa epithelial cell line GES-1, were obtained from ATCC (Manassas, VA, USA). Cells were cultured in RPMI 1640 medium with 10% fetal bovine serum and incubated at 37°C in an incubator containing 5% CO_2_.

PKM2 siRNA and the negative control were obtained from GenePharma (Shanghai, China). The cDNA of HIF-1*α* was cloned into the pcDNA3.1 plasmid (Invitrogen, Carlsbad, CA, USA) to establish the HIF-1*α* overexpression plasmid pcDNA3.1-HIF-1*α*. The BCL-6 overexpression plasmid pcDNA3.1-BCL6 was established by cloning the cDNA of BCL-6 into pcDNA3.1 plasmid. For transfection, cells reaching 80% confluence were transfected with siRNA or the recombinant plasmids using Lipofectamine 3000 (Invitrogen).

### 2.2. Cell Viability

Cell viability was detected using Cell Counting Kit-8 (CCK-8) (Beyotime, Shanghai, China) according to the manufacturer's instructions. The absorbance at a wavelength of 450 nm (OD_450_) was measured using an automicroplate reader.

### 2.3. Transwell Assay

For the invasion and migration assays, a 24-well transwell invasion chamber with an 8-*μ*m filter was used. Matrigel-coated filters (BD Biosciences, Bedford, MA, USA) were used for the cell invasion assay, and uncoated filters were used for the cell migration assay. Cells starved in serum-free DMEM were plated in the upper chambers (2 × 10^4^ cells/well). Then, DMEM with 10% fetal bovine serum was added to the lower chambers. After 24 hours of incubation, cells on the upper chambers were cleaned using a wet cotton swab. The migrated and invaded cells in the lower surface of the chamber were fixed and then stained with 0.5% crystal violet solution. Finally, the cells in each well (5 fields per well) were counted under a microscope.

### 2.4. Quantitative Real-Time PCR

RNA from the cancer cells was obtained using TRIzol reagent (Invitrogen). Reverse transcription was performed using a PrimeScript™ RT reagent kit (Takara, Dalian, China). Quantitative real-time PCR (RT-PCR) was employed to detect mRNA levels using a SYBR® Premix Ex Taq™ II kit (Takara) on an Applied Biosystems Prism 7500 Fast Sequence Detection System (Applied Biosystems, Foster City, CA, USA). *β*-Actin was used as an internal control.

The PCR primers were as follows: for PKM2, 5′-ACAGGAAGCCTCGTAAGGTC-3′ (sense) and 5′-CAGTCCCGGCTTCACTATGG-3′ (anti-sense); for E-cadherin, 5′-CCCACCACGTACAAGGGTC-3′ (sense) and 5′-CTGGGGTATTGGGGGCATC-3′ (anti-sense); for N-cadherin, 5′-GAGGAGTCAGTGAAGGAGTCA-3′ (sense) and 5′-GGCAAGTTGATTGGAGGGATG-3′ (anti-sense); for vimentin, 5′-AACTTAGGGGCGCTCTTGTCCC-3′ (sense) and 5′-CGCGCTGCTAGTTCTCAGTGCT-3′ (anti-sense); and for *β*-actin, 5′-ATTGCCGACAGGATGCAGAA-3′ and 5′-CAAGATCATTGCTCCTCCTGAGCGCA-3′. The results are represented as the relative mRNA level calculated using the 2^-*ΔΔ*CT^ method.

### 2.5. Western Blot

Cells were lysed with RIPA buffer (GeneCopoeia, Rockville, MD, USA), and total protein was isolated. Protein concentrations were detected using an enhanced BCA protein assay kit (Beyotime Biotechnology, Shanghai, China). The protein extracts were separated and then transferred electrophoretically onto PVDF membranes. Antibodies for rabbit polyclonal to PKM2 (ab85555, 1 : 2000), rabbit monoclonal to HIF-1*α* (ab51608, 1 : 1000), rabbit monoclonal to BCL-6 (ab172610, 1 : 1000), as well as the mouse monoclonal to *β*-actin (ab8226, 1 : 10000), were obtained from Abcam (Cambridge, MA, USA). The horseradish peroxidase-conjugated goat anti-rabbit IgG (ab205718, 1 : 5000) and horseradish peroxidase-conjugated goat anti-mouse IgG (ab205719, 1 : 5000) were purchased from Sigma (St. Louis, MO, USA). An ECL kit (Pierce Chemical, Rockford, IL, USA) was used for development. The results were normalized according to *β*-actin.

### 2.6. Statistical Analysis

Data were analyzed using the SPSS version 19.0 statistical software (SPSS Inc., Chicago, IL, USA) and are presented as the mean ± SD. Comparisons were performed using one-way ANOVA test. A *p* value < 0.05 was considered statistically significant.

## 3. Results

### 3.1. PKM2 Knockdown Suppressed Gastric Carcinoma Cell Invasion and Migration

**Figure 1:**
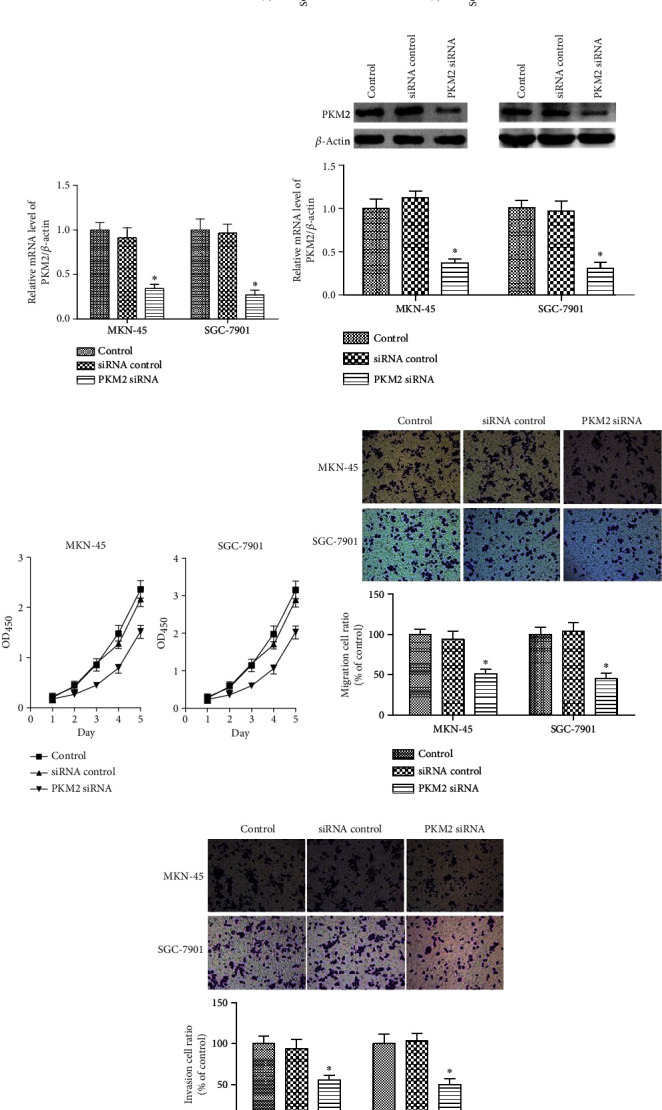
PKM2 knockdown suppressed human gastric cancer cell migration and invasion. (a) The PKM2 mRNA levels in human gastric mucosal epithelial cell GES-1 and gastric carcinoma cell lines were measured using qRT-PCR. ^∗^*p* < 0.05 versus GES-1 cells. (b) PKM2 protein expression was measured by western blot. ^∗^*p* < 0.05 versus GES-1 cells. MKN-45 and SGC-7901 cells were transfected with PKM2 siRNA. (c) The PKM2 mRNA levels were measured using qRT-PCR. (d) PKM2 protein expression was measured by western blot. (e) Cell viability in MKN-45and SGC-7901 cells was detected using CCK-8 assay. (f) Migration and (g) invasion were measured by transwell assay. The representative images (upper) and quantitative data (bottom) are shown. Data are expressed as the mean ± SD. ^∗^*p* < 0.05 versus the cell control group.

The mRNA and protein levels of PKM2 were determined in human gastric mucosal epithelial cell GES-1 and gastric carcinoma cell lines. We observed higher PKM2 mRNA in gastric carcinoma cell lines than in the GES-1 cells (Figure 1(a), *p* < 0.05). PKM2 protein expression was assayed by western blot, and the results showed higher protein levels of PKM2 in the gastric carcinoma cells than in the GES-1 cells (Figure 1(b), *p* < 0.05).

To determine the effect of PKM2 on cell migration and invasion, PKM2 expression was downregulated in MKN-45 and SGC-7901 cells via PKM2 siRNA transfection. The PKM2 siRNA significantly reduced the mRNA and protein levels of PKM2 (Figures 1(c) and 1(d), *p* < 0.05). Cell viability in MKN-45 and SGC-7901 cells was both significantly reduced by PKM2 knockdown (Figure 1(e), *p* < 0.05). The transwell assay indicated that cell migration decreased by 51.8% in MKN-45 cells and by 45.2% in SGC-7901 cells (Figure 1(f), *p* < 0.05). Also, PKM2 knockdown resulted in marked decreases in the rates of cell invasion in MKN-45 (55.7%) and SGC-7901 (50.3%) cells (Figure 1(g), *p* < 0.05).

### 3.2. PKM2 Knockdown Inhibited EMT Process of Gastric Carcinoma Cells

**Figure 2:**
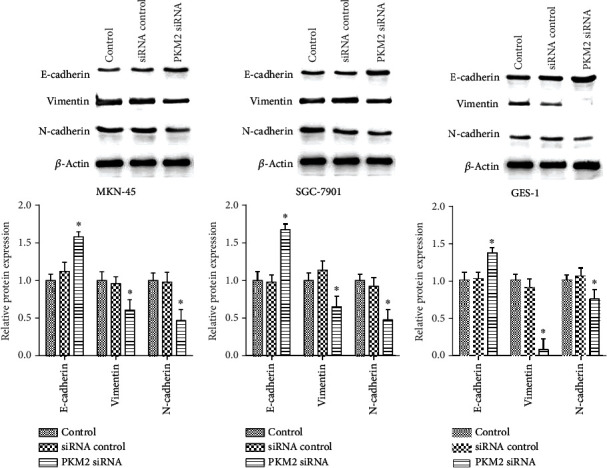
PKM2 knockdown suppressed epithelial-mesenchymal transition in human gastric cancer cells. Western blot assays were used to determine the protein expression in the human gastric carcinoma cell lines MKN-45 (a) and SGC-7901 (b), as well as human normal gastric mucosa epithelial cell line GES-1 (c). Data are expressed as the mean ± SD. ^∗^*p* < 0.05 versus the cell control group.

EMT is correlated with cancer cell invasion and migration [26]. To confirm the effect of PKM2 silencing on EMT in gastric carcinoma cells, the expression of EMT markers was detected by western blot. As shown in Figure 2, E-cadherin, N-cadherin, and vimentin expression did not differ between the control siRNA transfection group and the control group. PKM2 siRNA significantly increased expression of E-cadherin and decreased expression of N-cadherin and vimentin, compared with the control group (*p* < 0.05), in the human gastric carcinoma cell lines MKN-45 (Figure 2(a)) and SGC-7901 (Figure 2(b)). Besides, we also downregulated PKM2 by siRNA in human normal gastric mucosa epithelial cell line GES-1. It was also observed that PKM2 siRNA significantly increased expression of E-cadherin and decreased expression of N-cadherin and vimentin in GES-1 cells (Figure 2(c)).

### 3.3. PKM2 Knockdown Inhibited EMT, Migration, and Invasion of Gastric Carcinoma Cells via the HIF-1*α*/BCL-6 Signaling Pathway

**Figure 3:**
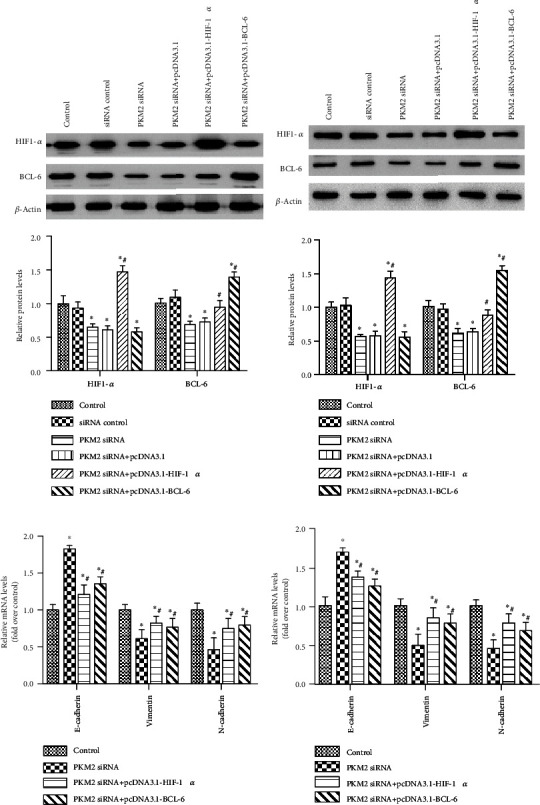
HIF-1*α*/BCL-6 upregulation reversed the effect of PKM2 knockdown on gastric carcinoma EMT. MKN-45 and SGC-7901 cells were transfected with control siRNA, PKM2 siRNA, PKM2 siRNA+pcDNA3.1, PKM2 siRNA+pcDNA3.1-HIF-1*α*, or PKM2 siRNA+pcDNA3.1-BCL-6. Western blot assays determined the protein expression of HIF-1*α* and BCL-6 in (a) MKN-45 and (b) SGC-7901 cells. The mRNA levels of EMT markers in (c) MKN-45 and (d) SGC-7901 cells were measured using qRT-PCR. Data are expressed as the mean ± SD. ^∗^*p* < 0.05 versus the cell control group. ^#^*p* < 0.05 versus the PKM2 siRNA group.

**Figure 4:**
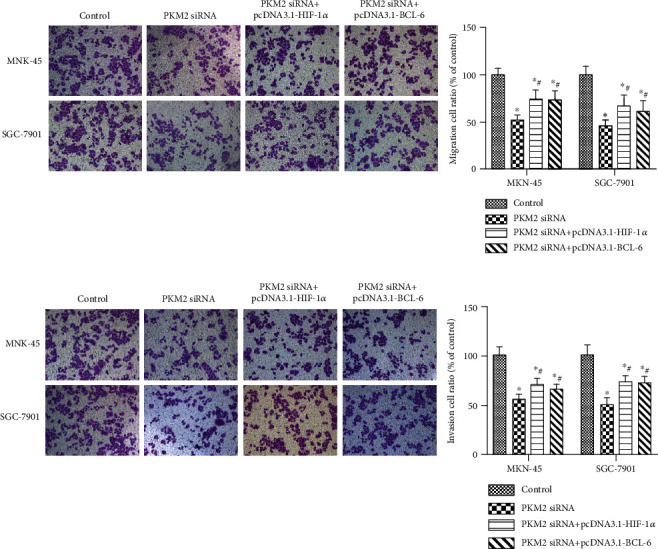
HIF-1*α*/BCL-6 upregulation reversed the effect of PKM2 knockdown on gastric carcinoma migration and invasion. MKN-45 and SGC-7901 cells were transfected with control siRNA, PKM2 siRNA, PKM2 siRNA+pcDNA3.1, PKM2 siRNA+pcDNA3.1-HIF-1*α*, or PKM2 siRNA+pcDNA3.1-BCL-6. Cell migration (a) and invasion (b) were measured by transwell assay. The representative images and quantitative data were shown. Data were expressed as the mean ± SD. ^∗^*p* < 0.05 versus the cell control group. ^#^*p* < 0.05 versus the PKM2 siRNA group.

A previous study demonstrated that PKM2 promotes angiogenesis of hypoxic pancreatic tumors by regulating HIF-1*α* expression [27]. HIF-1*α* and BCL-6 also play roles in gastric carcinoma [25, 28]. We thus assayed the expression levels of HIF-1*α* and BCL-6 in gastric carcinoma cells transfected with PKM2 siRNA. We observed significantly lower HIF-1*α* expression and lower BCL-6 expression in MKN-45 (Figure 3(a)) and SGC-7901 (Figure 3(b)) cells transfected with PKM2 siRNA, compared with the control group (*p* < 0.05). However, HIF-1*α* overexpression restored the downregulated expression of BCL-6 in gastric carcinoma cells (Figures 3(a) and 3(b), *p* < 0.05). HIF-1*α* overexpression also restored the upregulation of E-cadherin, as well as the downregulation of N-cadherin and vimentin in MKN-45 (Figure 3(c)) and SGC-7901 (Figure 3(d)) cells transfected with PKM2 siRNA. Additionally, PKM2 siRNA transfection inhibited cell migration (Figure 4(a)) and cell invasion (Figure 4(b)), both of which were reversed by HIF-1*α* overexpression. Similarly, BCL-6 overexpression restored the downregulated expression of BCL-6 in gastric carcinoma cells (Figures 3(a) and 3(b), *p* < 0.05). BCL-6 overexpression also restored the upregulation of E-cadherin, as well as the downregulation of N-cadherin and vimentin (Figures 3(c) and 3(d)), and restored the cell migration (Figure 4(a)) and cell invasion (Figure 4(b)) in both MKN-45 and SGC-7901 cells transfected with PKM2 siRNA. But BCL-6 overexpression affected little on the expression of HIF-1*α* (Figures 3(a) and 3(b)).

## 4. Discussion

During the last step of glycolysis, the PKM2 enzyme catalyzes phosphorylation of phosphoenolpyruvate to pyruvate and generates ATP [27]. PKM2 is upregulated in many cancers, including ovarian cancer [29], colorectal cancer [30], myeloma [10], and cervical carcinoma [31]. PKM2 was regarded as an adverse prognostic factor for gastric carcinoma [32]. PKM2 expression regulates cancer-specific metabolism to promote gastric carcinoma [33]. In thyroid cancer cells, the knockdown of PKM2 inhibited cell invasion and migration [34]. In previous reports, it was demonstrated that PKM2 was upregulated in gastric cancer tissues and cell lines [16, 35, 36]. Our present study also confirmed that PKM2 was highly expressed in gastric carcinoma cells. We also demonstrated that PKM2 knockdown suppressed gastric cancer cell migration and invasion. This was in accordance with a previous study, in which the authors showed that PKM2 was involved in the regulation of cell proliferation, migration, and invasion in gastric carcinoma cells [16]. However, we also observed that cell viability in MKN-45 and SGC-7901 cells were both significantly reduced by PKM2 knockdown. The decreased cell viability might also be involved in the effect of PMK2 knockdown on cell migration and invasion.

PKM2 is a target gene of HIF-1*α* and participates in a positive feedback loop that promotes HIF-1 transactivation and reprograms glucose metabolism in cancer cells [37]. In prostate cancer, HIF-1*α* mediates expression of PKM2 [38]. Consistent with previous reports, our study confirmed that PKM2 silencing markedly impeded expression of HIF-1*α*. HIF-1*α* is an oxygen-sensitive regulator that influences cell survival, proliferation, angiogenesis, and metabolism [37]. A rapidly growing body of evidence indicates that HIF-1*α* enhances cancer development [39]. HIF-1*α* is involved in SGC-7901 cell growth, apoptosis, and tumor angiogenesis [28]. In the present study, we confirmed that HIF-1*α* participated in the effects of PKM2-silencing on MKN-45 and SGC-7901 cell biology. Whether HIF-1*α* mediates PKM2 expression in gastric carcinoma remains unclear. Our future study will focus on the relationship between HIF-1*α* and PKM2.

BCL-6 is a zinc finger-like structure protein and intranuclear transcriptional repressor involved in regulating organ development and cell proliferation and differentiation [40]. BCL-6 also is associated with gastric carcinoma cell proliferation and invasion [25]. In the present study, BCL-6 overexpression reversed the effect of PKM2 knockdown on the invasion, migration, and EMT in gastric carcinoma cell, suggesting that BCL-6 might play a fundamental role in the function of PKM2 silencing in gastric carcinoma cell invasion, migration, and EMT.

## 5. Conclusions

These data demonstrate that PKM2 silencing suppressed gastric carcinoma cell migration and invasion via the HIF-1*α*/BCL-6 signal pathway. Our findings further the understanding of the function and molecular mechanism of PKM2 in gastric carcinoma cells. However, in vivo experiments and studies of tissue from patients with gastric carcinoma will help clarify the function of PKM2 in gastric carcinoma.

## Data Availability

The datasets used and/or analyzed during the current study are available from the corresponding author on reasonable request.

## Abbreviations

PKM2: Pyruvate kinase M2
EMT: Epithelial-to-mesenchymal transition
BCL-6: B-cell lymphoma 6
HIF-1*α*: Hypoxia-inducible factor alpha.
